# Pancreatic Cancer, Gut Microbiota, and Therapeutic Efficacy

**DOI:** 10.7150/jca.37445

**Published:** 2020-02-20

**Authors:** Xiang Zhang, Qiaofei Liu, Quan Liao, Yupei Zhao

**Affiliations:** Department of General Surgery, Peking Union Medical College Hospital, Chinese Academy of Medical Sciences & Peking Union Medical College, Beijing 100730, China

**Keywords:** gut microbiota, pancreatic cancer, chemotherapy, immunotherapy, tumor microenvironment

## Abstract

Pancreatic cancer remains one of the leading causes of cancer-related death worldwide and has a poor prognosis. Current treatment relies on surgical resection and adjuvant therapies. The gut microbiota plays important roles in metabolism and immunomodulation. Accumulating evidence has implied that the gut microbiota is involved in the metabolism of chemotherapeutic drugs and the tumor microenvironment (TME), which could affect the efficacy of both conventional chemotherapy and immunotherapy for pancreatic cancer. Herein, we comprehensively reviewed the history and highlights of the interactions among pancreatic cancer, the gut microbiota and therapeutic efficacy and showed the promising future of manipulating the gut microbiota to improve clinical outcomes of pancreatic cancer.

## Introduction

Pancreatic ductal adenocarcinoma (PDAC) accounts for more than 85% of pancreatic cancer cases. PDAC is still one of the most devastating malignancies, with a 5-year overall survival of less than 10%. Since less than 20% of PDAC patients have the opportunity for surgical resection, chemotherapy remains the main treatment option. Since its approval by the Food and Drug Administration (FDA) in 1996, gemcitabine has been actively used to treat PDAC that has progressed in the extensive desmoplastic microenvironment surrounding the few remaining cancer cells, but chemoresistance and reduced sensitivity are often acquired during multiple weeks of chemotherapy cycles 1. This hypovascular and highly desmoplastic tumor tissue leads to poor drug delivery and ineffectiveness of cytotoxic agents 2. However, morphological characteristics are only partly responsible for resistance. Great efforts have been made to solve this difficult problem. Gemcitabine-based combined therapies, tumor microenvironment (TME)-targeting strategies and immunotherapy are being developed to overcome drug resistance and ineffectiveness in PDAC 3.

The gut microbiota has been recognized as a considerable ecosystem, comprising over 10^14^ microorganisms, and it encodes far more genes than the human body 4,5. Increasing evidence links the microbiota, cancer progression and therapeutic responses 5. Several population-based studies indicate that oral pathogenic microorganisms are associated with an increased risk of PDAC 6-9. This may be due to systemic inflammatory and immune responses induced by some specific bacteria and bacterial metabolites, which could regulate cancer-related immunomodulation. Recent data have suggested that the gut microbiota play critical roles in human pancreatic diseases, including pancreatitis and PDAC 10. Large numbers of bacterial metabolites may participate in the regulation of pancreatic carcinogenesis, the immune system and therapeutic resistance. Intratumoral bacteria have also been found in the TME of PDAC. The interactions between the host microbiota and therapeutic efficacy may be an important breakthrough in understanding the altered efficacy of chemotherapeutic agents and immunotherapies towards PDAC.

To date, common techniques used to assess microbial communities, e.g., 16S ribosomal RNA (rRNA) sequencing, metagenomic sequencing, quantitative PCR and culturomics, have greatly expanded our knowledge of the diversity and multifunctionality of the microbiota. Herein, we summarize the history and progression of the reciprocal interactions between the gut microbiota and therapeutic efficacy in PDAC and discuss how these developments have paved the way to improve patient survival.

## Therapeutic Dilemma of Pancreatic Cancer

Difficult issues regarding chemotherapy and immunotherapy of advanced-stage PDAC mainly concentrated on the heterogeneous efficacy of individuals who had the same histopathologic tumor characteristics. Essential factors determining drug sensitivity include pancreatic cancer cells and their surrounding components, such as the extracellular matrix, immune cells (e.g., tumor-associated macrophages (TAMs), myeloid-derived suppressor cells (MDSCs), tumor-associated neutrophils), cancer-associated fibroblasts, pancreatic stellate cells and cancer stem cells, all of which impair the normoxic microenvironment of the pancreas 11,12. The desmoplastic stroma-rich microenvironment restricts intratumor blood supply and drug delivery. Hypoxia and cancer somatic mutations also contribute to therapeutic resistance 13. Recent achievements have also promoted clinical research on the TME to increase pharmaceutic penetration into tumor tissue. However, clinical trials of stroma-targeting compounds plus gemcitabine have failed to improve patient survival in metastatic PDAC 14. PDAC responds poorly to a single application of immune checkpoint inhibitors (ICIs), e.g., anti-programmed cell death protein 1 (PD)-1/anti-PD-L1, anti-CTLA-4, and anti-LAG-3 15,16. In most cases, the agents do not work, and their limited effects are offset or compensated under the coordination and crosstalk of multiple suppressive mechanisms of the host immune system.

Recent studies have emphasized the difference in the gut microbiota between cancer patients and healthy individuals. The microbiota of the duodenal mucosa in PDAC patients and healthy controls shared similar species in one study. However, duodenal samples of PDAC were characterized by enrichment with, for example, *Acinetobacter*, *Aquabacterium*, *Oceanobacillus* and *Rahnella* 17. Limitations exist in determining whether microbiota alterations contribute to tumor progression, and whether the altered host microbiota is merely a concomitant manifestation remains elusive. A considerable proportion of nonantibiotics also influence the growth of bacterial species 18. A resistant starch diet promoted a decrease in tumor progression in PDAC xenograft mice, which was associated with a reduction in proinflammatory fecal microbiota 19.

The mechanisms of the TME in PDAC that affect the efficacy of therapies remain largely unknown and require further exploration. Recently, accumulating evidence has shown that the gut microbiota has a potential role in regulating cancer-related immunomodulation and treatment, presenting new targets to improve therapeutic efficacy.

## The Role of the Gut Microbiota in Chemotherapy and Immunotherapy

Based on previous publications in the past decade, the gut microbiota may lead to altered efficacy of pharmacotherapeutics in cancer treatment (Table 1). Although the explicit role of the microbiota in host immunity, especially in the tumor-specific TME, remains unclear, the interactions between tumor control and gut microbiota have become more intertwined than ever before 20,21. In drug-free conditions, interactions between the host and the gut microbiota involve the mucus layer, epithelial cells, dendritic cells (DCs) and immune cells 22-25. In cancer patients, summarized evidence implies a bidirectional relationship between the host microbiota and various types of cancer therapies.

### Conventional Chemotherapy

Previous evidence has demonstrated that both cancer cells and the related TME components participate in cancer development and treatment adaptation. Recent studies revealed that the gut microbiota may contribute to the efficacy of conventional chemotherapeutic agents through drug metabolism, biotransformation and immune regulation 50.

The gut microbiota has favorable effects on chemotherapy *in vitro* and *in vivo*. Previously, the clinical potential for microbial therapeutic use was indicated by the synergism of *Salmonella typhimurium* in mouse models of PDAC treated with gemcitabine and bevacizumab 51. The culture supernatant of *Lactobacillus plantarum* was shown to have positive effects on improving the chemosensitivity of 5-fluorouracil (5-FU) in colorectal cancer (CRC) cells by inhibiting cancer stem-like cell formation 32. An intact commensal microbiota, as a modulator in the TME, is required for optimal anticancer drug responses via the functional maintenance of myeloid-derived cells through Toll-like receptors (TLRs) 35. Attenuated cytotoxic effects of oxaliplatin were observed in germ-free and antibiotic-treated subcutaneous tumor-bearing animals. An intact microbiota was required for priming tumor-associated myeloid cells that produce reactive oxygen species, which are important for oxaliplatin cytotoxicity 35. In tumor-bearing mouse models treated with cyclophosphamide (CTX), the gut microbiota promoted an adaptive immune response to restore antitumor efficacy 36. CD8^+^ T cells perform important duties in the adaptive antitumor immune response. The commensal bacterial species *Enterococcus hirae* (*E. hirae*) and *Barnesiella intestinihominis* were identified in CTX-induced immunomodulation, with altered TME and enhanced anticancer CTL responses. These bacteria were capable of partially restoring host T cell responses and improving the therapeutic efficacy of CTX or other alkylating agents 31. Interestingly, translocation of some intestinal bacterial species (gram-positive) into secondary lymphoid organs was observed in response to CTX 36. Translocated bacteria enhanced the bioactivity of adoptively transferred CD8^+^ T cells and innate immunity 52. In addition, chemotherapeutic platinum agents were also found to induce bacterial translocation across the intestinal barrier and activate T helper 1 (Th1) memory responses 53.

However, microbiota, e.g. *Fusobacterium nucleatum* in CRC, were found to promote chemoresistant status 26,30,54. A cocktail of antibiotics increased Proteobacteria and reduced 5-FU efficacy in CRC mice 27. Furthermore, bacterial metabolism was reported to affect the efficacy of CPT, 5-FU and 5-fluoro-2′-deoxyuridine (FUDR) against *Caenorhabditis elegans* 55. Prior studies investigating chemotherapy-related microbiota alterations revealed a severe imbalance in microbial composition and function, leading to intestinal dysbiosis 56. Gemcitabine-treated xenografted mouse models of PDAC revealed proinflammatory alterations in the fecal microbiota, with an increase in *Proteobacteria* and *Verrucomicrobia* and a decrease in *Firmicutes* and *Bacteroidetes*, as well as activation of the NF-κB inflammatory pathway in tumor tissues 57. The latest multicenter clinical trial revealed that FOLFIRINOX chemotherapy for PDAC led to longer survival than standard gemcitabine adjuvant therapy at the expense of a higher incidence of toxic events, e.g., diarrhea and nausea 58. The side effect of CPT-11 (irinotecan) chemotherapy, diarrhea, occurs in many cancer cases. CPT-11's inactive metabolite SN-38G (transferred by carboxylesterase) was restored to its active form by β‑glucuronidase‑expressing bacteria in the gut, leading to enteral release of the active SN-38 metabolite and severe diarrhea 59. Using streptomycin reduced enteral epithelium absorption of SN-38 and decreased carboxylesterase activity 60. Thus, chemotherapeutics induce efficacy, alter the microbiota and cause toxicity, which presents a challenge in achieving optimal anticancer effects and reducing side effects by manipulating the gut microbiota. Collectively, current findings have elucidated the complex influences of the gut microbiota on exogenous drugs and endogenous responses.

### Immunotherapy

Unlike conventional chemotherapy, immunotherapy targets the immune microenvironment beyond the tumor cells. One of the crucial mediators linking the microbiota to the immune response is TLRs, which are categorized as cytoplasmic pattern recognition receptors. TLR4 binding to bacterial lipopolysaccharides triggers *in situ* and systemic inflammation. A recent study reported that microbial stimulation of cancer cells overexpressed cathepsin K, which promoted immunosuppressive M2 TAM polarization through the TLR4-mTOR pathway 61. Attenuated immunocyte-targeting bacterium *Listeria monocytogenes* modified the suppressive cancer microenvironment by reducing peripheral and intratumor MDSCs and repolarizing the TAM subpopulation from the M2 phenotype to the antitumor M1 phenotype 62,63. *Bacteroides* species activate Th1 immune responses and promote the maturation of DCs within tumors 49. *Faecalibacterium* and butyrate-producing bacteria were associated with Foxp3+ regulatory T cell (Treg) accumulation in the gut, whereas* Bifidobacterium adolescentis*,* Parabacteroides merdae*, *Collinsella aerofaciens* (*C. aerofaciens*), and* Enterococcus faecium* (*E. faecium*) inhibited Tregs in humans 40,45. Tregs expressing TLR2 could suppress immune responses in cancer treatment 45,64. A genetically engineered mouse model (GEMM) has provided us with a more accurate imitation of human cancer progression and natural TME components. Sethi and coworkers found that gut microbial depletion via oral antibiotics caused a significant decrease in pancreatic cancer burden and an activated anticancer immune response in GEMM 65. C57BL/6J wild-type mice, Rag1 knockout mice lacking mature T and B lymphocytes and Kras^G12D/+^, Trp53^R172H/+^, Pdx-1^cre^ (KPC) mice were comparatively analyzed. Microbial ablation led to significant changes in critical components of the TME, presenting as increased populations of IFNγ^+^CD4^+^CD3^+^ T helper 1 cells and IFNγ^+^CD8^+^CD3^+^ cytotoxic T cell 1 (Tc1) population, with a simultaneous decrease in protumor immune cells 65. These inflammatory cells are usually cancer-associated and infiltrate into the TME, which may influence therapeutic efficacy.

Attention has been paid to ICIs in the clinic. Immunotherapy targeting PD-1 has emerged as an effective strategy for the treatment of several cancers. Accumulating data revealed that T cell infiltration and variable immune regulators, including the gut microbiota, were associated with PD-1/PD-L1 blockade in patients, with beneficial outcomes for some cancers 66-69. Among melanoma patients, fecal microbiota analysis identified a favorable abundance of the Ruminococcaceae family and *Clostridiales* in anti-PD-L1 responders with enhanced antitumor immune responses, whereas nonresponders were enriched with *Bacteroidales* 41. Ruminococcaceae bacteria in the gut were associated with a higher density of peripheral and infiltrating effector T cells. A higher relative abundance of gut bacterial species, including *Bifidobacterium longum*, *C. aerofaciens*, *E. faecium* and *Bacteroides caccae*, was detected in immunotherapeutic responders versus nonresponders among metastatic melanoma patients 40,46. Interpatient heterogeneity may be derived from differences in individual microbial composition. A favorable composition of commensal microbiota in a melanoma mouse model resulted in enhanced antitumor immunity and improved therapeutic activity of anti-PD-L1 treatment 48. Hereinto, *Bifidobacterium* in mice directly stimulated DCs and induced the maturation of DCs. These novel findings indicated the potential of the gut microbiota for regulating host responses towards immunotherapies. Routy et al. 43 observed that antibiotic treatment suppressed the clinical benefit of ICIs (overall survival and progression-free survival) when treating epithelial tumor (non-small cell lung cancer, renal cell carcinoma and urothelial carcinoma) patients. Metagenomic analysis of patient fecal samples revealed the correlation between ICI responses and *Akkermansia muciniphila* (*A. muciniphila*) dysbiosis, which showed a restoration of PD-1 blockade resistance in mouse tumor models after oral administration. Intestinal *A. muciniphila* increased the recruitment of CCR9^+^CXCR3^+^CD4^+^ T cells in the tumor bed, suggesting that future immunotherapeutic targets could manipulate the gut microbiota in individuals with cancer. Furthermore, *in vitro*, *A. muciniphila* and *E. hirae* stimulated DCs to secrete interleukin-12 (IL-12), which is the crucial cytokine for Th1 cell differentiation and function 43,70. However, other clinical observations in non-small-cell lung cancer showed no beneficial impact of antibiotics on anti-PD-1 therapy 39,44,47.

Briefly, host immunity and TME always play crucial roles in microbiota-modified therapeutic responses. Specific gut microbiota have the potential to predict the efficacy of certain kinds of immunotherapies, and colonization of tumor-specific bacteria has been found to play regulatory roles in the antitumor effects of immune-targeting treatment. The presence of microbiota-derived mediating factors and host variability will create a heterogeneous local TME and relevant alterations in systemic communication.

## Intratumor Microbiota of Pancreatic Cancer

Recent advances have begun to elucidate the potential roles of intratumoral microorganisms in anticancer therapeutics, e.g., pancreatic cancer 71. Based on conventional speculation, the pancreas tissue has no direct contact with the gut microbiota from both a clinical and anatomical perspective. Many clinicians believe that pancreatic tissue is germ free; otherwise, the patient or individual may be infected and will have a fever of pancreatic origin. Notably, recent studies in mice and humans found that bacteria exist not only in pancreatic tumor tissues but also in normal pancreatic tissues. Nevertheless, cancerous tissue harbors an increased abundance of microorganisms 42. Geller et al. 29 reported that 15% (3/20) of normal pancreatic tissues contain bacterial DNA via qPCR detection. In both PDAC and noncancer patients, similar bacterial profiles were detected at different sites of the pancreas and duodenum tissues within the same individual, suggesting that intrapancreatic bacteria may migrate from the surrounding gut tract across the intestinal wall 72.

The association of gut bacteria and tumor tissue has been previously reported, such as *Helicobacter pylori* and gastric cancer, *Salmonella typhi* and gallbladder cancer, and altered bacterial species and CRC 73-75. These microorganisms within tumors may stimulate host immune responses and generate beneficial or disruptive impacts on anticancer therapy, as determined by pharmacological mechanisms, as well as the major response pathways 53. Some human solid tumors were found to be infected with *Mycoplasma hyorhinis* (*M. hyorhinis*), which was shown to have a relationship with gemcitabine drug resistance 76. *M. hyorhinis* infection led to weakened therapeutic efficacy of gemcitabine treatment via the microbial enzyme cytidine deaminase (CDD). Gemcitabine (2',2'-difluorodeoxycytidine) was metabolized into its inactive deaminated form, 2',2'-difluorodeoxyuridine (dFdU), in *M. hyorhinis*-infected conditioned cultures 29,34. In human PDAC, 76% (86/113) of the tissue samples exhibited the presence of bacteria from the intratumoral *Gammaproteobacteria* class, which contain the enzyme CDD; this enzyme was indicated to be responsible for the ineffectiveness of gemcitabine in PDAC 29,77. Bacterial CDD exhibits two different forms: long CDD (CDD_L_) and short CDD (CDD_S_). The expression of the resistance-related isoform CDD_L_ led to the metabolism of gemcitabine 34. Moreover, high-performance liquid chromatography and mass spectrometry identified *Escherichia coli* (*E. coli*)-induced chemical structure modification of gemcitabine, fludarabine, cladribine and CB1954 33. Nonpathogenic *E. coli* lowered the cytotoxicity of gemcitabine *in vitro* and in subcutaneous colorectal carcinoma models containing intratumoral bacteria 33.

The *Fusobacterium* species, a group of oral bacteria, were initially detected in PDAC tissues by Mitsuhashi et al. 78 and were found to be independently associated with a worse patient survival probability. The intrapancreatic abundance of *Fusobacterium* species was found to be relatively higher in PDAC subjects than in noncancer controls 72. *Fusobacterium nucleatum* elicited chemoresistance to 5-FU and oxaliplatin in CRC, targeting TLR4 and MyD88 immune signaling and activating the cancer autophagy pathway via downregulation of miR-18a* and miR-4802 30. TLR4/MyD88 signaling was also previously associated with chemoresistance to paclitaxel in ovarian cancer 79. With respect to PDAC, the desmoplastic response induced by cancer cells was dependent on MyD88 signaling to create an immunosuppressive TME, suggesting the potential impact of *Fusobacterium* species on the chemoresistance of PDAC 80.

Bacteria are capable of translocating from the gut to the pancreas in mice and influencing the PDAC microenvironment 42. Moreover, local bacteria in the human pancreas that migrate from the gut play a specific role in cancer progression and treatment and, to some extent, may be a neglected component of the traditionally defined TME. In the complex TME of PDAC, TAMs and MDSCs constituted the leading population of infiltrated immunosuppressive components 81. Antibiotic-mediated removal of intrapancreatic bacteria was associated with immune remodeling of the TME in PDAC-bearing KPC mice. The observed cellular events mainly involve (1) reduced MDSCs; (2) increased M1-polarized TAMs (repolarized from the M2 phenotype); and (3) immune-promoting activation of CD4^+^ and CD8^+^ T cells with elevated expression of T-BET, TNF-α, CD38, PD-1 and CD44 42. The study revealed that antibiotics plus PD-1 inhibition generated synergistic anticancer efficacy, with enhanced intratumoral T cell activation. However, these mouse-based preclinical results for PDAC are in contrast to the abovementioned research by Routy et al. 43 on multiple solid tumors of nondigestive systems. This indicates that distinct microbiota alterations may cause bacteria-specific effects on the corresponding organs 82. In addition, enteral and parenteral translocation, local and systemic responses, and the predominant microflora will determine the final direction of therapeutic efficacy.

Recently, intratumor microbiota were further confirmed to be highly predictive of long-term survivorship in PDAC patients, and experimental evidence suggested that intratumor microbiota were modified by the gut microbiota 83. Transplantation of fecal microbiota from PDAC long-term survivors restored a treatment-friendly immune microenvironment in tumor mice. These findings highlight the role of microbiota as a promising therapeutic intermediate for PDAC.

**Figure 1:**
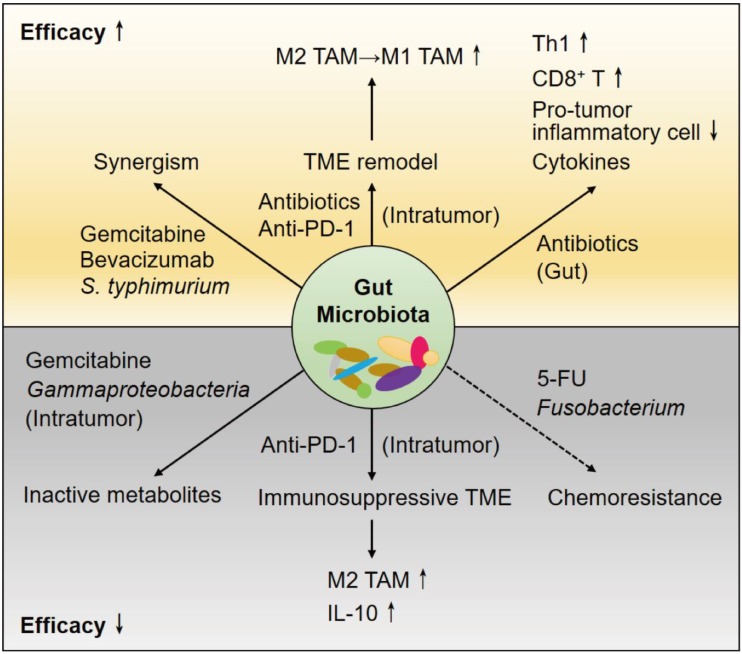
The gut microbiota and therapeutic effects in the cancerous pancreas. The gut microbiota has impacts on host immunity, the tumor microenvironment (TME) of PDAC and the effects of therapeutic agents. Antibiotics cause depletion of some bacterial species, leading to augmented antitumor responses. The cytotoxic effects of chemotherapeutics are attenuated by the microbiota in the gut and within the pancreas or tumor. Immune checkpoint inhibitors (ICIs) usually result in poor responses in immunosuppressive patients with PDAC. Antibiotics induce TME remodeling and enhance anti-PD-1 efficacy. In addition, the components and functions of the microbiota are modified by the host immune response and therapeutic drugs.

To date, continued attempts to overcome cancer chemoresistance and immune tolerance have not attained beneficial overall survival for PDAC patients, even after R0 resection with pathologically tumor-free surgical margins. The gut microbiota has been recognized as a crucial mediator in the TME of PDAC; thus, the crosstalk among these regulators becomes more complex and pluralistic (Figure 1). Considering that microorganisms outnumber human somatic cells, further use of antibiotics to shape the gut microbiome is likely to overcome the hurdles.

## Microbiota Transplantation

The concept of fecal microbiota transplantation (FMT), which originates from the fourth century in ancient China, has overcome challenges and instigated wide discussion both technologically and theoretically 84. With the increasing use of FMT, fecal therapy has become a promising strategy in diverse human diseases, as shown in novel clinical reports on epilepsy, hepatic encephalopathy and metabolic syndrome 85-87. Le Bastard et al. 88 reported that ampicillin- and/or 5-FU-pretreated C57BL/6J mice exhibited a critical alteration of bacterial species distribution and presented functional disruption, which was corrected by receiving FMT. This may be helpful to prevent and treat several gastrointestinal side effects caused by chemotherapy, immunotherapy and antibiotics. Moreover, the first successful attempt at treating refractory ICI-associated colitis with FMT was recently reported 89. The two enrolled patients achieved improvement in clinical symptoms during the follow-up. Recently, Tanoue et al. 90 separated 11 bacterial strains from the fecal microbiota of healthy volunteers with the ability to induce interferon-gamma (IFN-γ)-expressing CD8^+^ T cell accumulation in the intestine. The 11 isolated strains showed effectiveness in spontaneous ICI treatment and dependent tumor inhibition via enhanced CD8^+^ T cell antitumor immunity. The reported mixture of 11 strains, which were mostly rare and low-abundance species among normal human microbiota, showed great therapeutic potential for resistance to chemotherapy and immunotherapy for widespread cancer types.

Given its success with metastatic melanoma, FMT could allow PDAC patients to seek improved prognosis safely 91,92. Although it remains unknown whether FMT could restrict oncogenesis in humans, microbiota transplantation seems to be a promising approach to further manipulate microbial composition and function to enhance host anticancer immunity and to improve resistance and ineffectiveness in cancer patients with relatively short survival. These gut and intratumoral microflora will become future targets to overcome pancreatic oncogenesis and immunosuppression.

## Microbial Markers for Therapeutics

Distinct responses to chemotherapy and immunotherapy in cancer patients have provoked robust interest in identifying useful biomarkers to optimize patient selection and management. Fecal sample analysis using 16S rRNA sequencing has provided evidence that *Fusobacterium nucleatum* is related to the chemoresistance of CRC, as well as other specifically chemotherapy-associated bacterial strains 28. This may be an optimal microbial marker candidate during anticancer treatment. Chemotherapy-induced gastrointestinal mucositis, which is clinically manifested as diarrhea, abdominal pain, malnutrition and bacteremia, remains an unpleasant side effect of chemotherapeutic agents 93. A higher abundance of baseline gut microbiota (*Faecalibacterium* genus and other Firmicutes) predicted favorable clinical responses among ipilimumab-treated individuals with metastatic melanoma but increased onset of ipilimumab-related colitis 45. Inflammation can be either a cause or a consequence of the gut microbiota. A hypothesis concerning commensal intestinal bacteria, chemotherapy and mucositis has been proposed with five potential aspects: inflammatory process and oxidative stress, intestinal permeability, mucus layer constituents, epithelial repair and immune effector molecules 94. From the perspective of mechanism and etiology, microbial shifts may also reflect the development of intestinal mucositis.

Use of the gut microbiota as a marker for drug efficacy and side effects should be qualified with significantly altered abundance and differential function between postoperative adaptation and drug response. Some issues should be urgently addressed, such as the specificity of biomarker bacteria, the accuracy of comparative studies and how the remote prediction of bacteria works 95. Future studies will discuss potential biomarkers for monitoring treatment responses, explore possible mechanisms underlying resistance and guide clinical dosage adjustment.

## Perspectives

Altered therapeutic efficacy resulting from the host microbiota typically changes in terms of multiple parameters. The quantitative approach used to evaluate the contributions of the microbiota, as previously described in the study of brivudine metabolism, will be appreciated for its role in the effort against drug tolerance in PDAC 96. Pharmacomicrobiomics has been frequently used in recent years to investigate the interactions between the microbiota and drugs 97,98. Current microbe-based anticancer therapy has attracted increasing attention from clinicians and oncologists and has inspired them to increase their efforts to control immunoregulation, therapeutic efficacy and drug safety 99.

## Conclusions

It is almost impossible for the human body to be absolutely sterile or germ free. All therapeutic strategies and host responses will be directly or indirectly influenced by the microbiota. Future studies should focus on not only the tumor itself but also the treatment dilemma, which might have a long-term influence on the ecosystem in the gastrointestinal tract 4. We reviewed the potential association between the gut microbiota and therapy regimens and highlighted the microbiota-therapeutic interactions in PDAC, which is of significance for patients lacking effective anticancer drugs.

The reciprocal interactions between the gut microbiota and cancer therapies are complicated and are cancer-dependent, therapy-dependent, and even tumor stage-dependent, leading to the paradox that in some cancers, the gut microbiota is a prerequisite to maintain therapeutic efficacy; however, in other cancers, depletion of the gut microbiota significantly improves efficacy. A large amount of exploratory work is needed to comprehensively understand the role of the microbiota in influencing therapeutic responses in PDAC. We suggested that modified dietary supplements, FMT and the application of certain antibiotics would have an impact on augmented drug efficacy, reduced toxicity and restricted PDAC recurrence and metastasis 100,101. Using 16S rRNA identification, metagenomics analyses and other high-throughput techniques enables clinicians to monitor therapeutic efficacy before the development of invasiveness and to provide a possible salvaged target to make paradigm shifts to the current first-line regimens.

## Figures and Tables

**Table 1:** Preclinical and clinical studies on the microbiota and therapeutic efficacy against solid tumors in the past decade.

Studies	Therapeutic drugs or targets	Microbiota or microbial intervention	Efficacy	Mechanisms
**Chemotherapy**				
Zhang et al. 26	5-Fluorouracil	*Fusobacterium nucleatum*	Nonbeneficial	Induce BIRC3 expression via theTLR4/NF-κB pathway
Yuan et al. 27	5-Fluorouracil	Antibiotics increase Proteobacteria	Nonbeneficial	-
Deng et al. 28	Tegafur plus oxaliplatin	*Fusobacterium nucleatum*	Nonbeneficial	-
Geller et al. 29	Gemcitabine	Gammaproteobacteria	Nonbeneficial	Bacterial CDD inactivates gemcitabine
Yu et al. 30	5-Fluorouracil/oxaliplatin	*Fusobacterium nucleatum*	Nonbeneficial	Activate TLR4/MyD88 signaling and autophagy
Daillère et al. 31	Cyclophosphamide	*Enterococcus hirae*	Beneficial	Translocation increases CD8/Treg ratio within tumor
		*Barnesiella intestinihominis*	Beneficial	Increase IFN-γ^+^ γδ^+^ T cells within tumor
An and Ha 32	5-Fluorouracil	*Lactobacillus plantarum*	Beneficial	Decrease cancer stem-like cells
Lehouritis et al. 33	Fludarabine phosphate/CB1954	*E. coli* Nissle 1917, *Listeria welshimeri* Serovar 6B SLCC5334	Beneficial	Drug modification
	Gemcitabine/cladribine		Nonbeneficial	-
Vande et al. 34	Gemcitabine	*Mycoplasma hyorhinis*	Nonbeneficial	Bacterial CDD and nucleoside phosphorylase decrease cytostatic activity
Iida et al. 35	Oxaliplatin/cisplatin	Antibiotic treatment	Nonbeneficial	Reduce myeloid-cell ROS
Viaud et al. 36	Cyclophosphamide	*Lactobacillus johnsonii*, *Lactobacillus murinus*, *Enterococcus hirae*	Beneficial	Induce bacterial translocation, which stimulates pathogenic Th17 and memory Th1 immune responses
**Immunotherapy (Underlined microbiota were involved in the mechanisms)**				
Zheng et al. 37	PD-1	*Akkermansia muciniphila*, *Ruminococcaceae* spp.	Beneficial	-
		Proteobacteria	Nonbeneficial	-
Peters et al. 38	PD-1/CTLA-4	*Faecalibacterium prausnitzii*, *Coprococcus eutactus*, *Prevotella stercorea*, *Streptococcus sanguinis*, *Streptococcus anginosus*, *Lachnospiraceae bacterium 3 1 46FAA*	Beneficial	-
		*Bacteroides ovatus*, *Bacteroides dorei*, *Bacteroides massiliensis*, *Ruminococcus gnavus*, *Blautia producta*	Nonbeneficial	-
Zhao et al. 39	PD-1	Antibiotic treatment	Nonbeneficial	-
Matson et al. 40	PD-1	*Enterococcus faecium*, *Collinsella aerofaciens*, *Bifidobacterium adolescentis*, *Klebsiella pneumoniae*, *Veillonella parvula*, *Parabacteroides merdae*, *Lactobacillus* sp., *Bifidobacterium longum*	Beneficial	Decrease Tregs
		*Ruminococcus obeum*, *Roseburia intestinalis*	Nonbeneficial	-
Gopalakrishnan et al. 41	PD-1	Ruminococcaceae/Faecalibacterium	Beneficial	Increase peripheral and infiltrating effector T cells
		Bacteroidales	Nonbeneficial	-
Pushalkar et al. 42	PD-1	Intratumoral microbiota	Nonbeneficial	Induce immunosuppressive tumor microenvironment
Routy et al. 43	PD-1	*Akkermansiacea muciniphila*, *Enterococcus hirae*, *Alistipes indistinctus*	Beneficial	Increase CD4+ central memory T cells, IL-12 secretion of DC, and intratumor CD4/Foxp3 ratios and elicit Th1 immune responses
Derosa et al. 44	PD-1/CTLA-4	Antibiotic treatment	Nonbeneficial	-
Chaput et al. 45	CTLA-4	*Faecalibacterium prausnitzii*, butyrate-producing bacterium, *Gemmiger formicilis*	Beneficial	Induce Tregs in the gut
		Bacteroidetes/Bacteroides	Nonbeneficial	-
Frankel et al. 46	PD-1/CTLA-4	*Bacteroides caccae*, *Streptococcus parasanguinis*, *aecalibacterium prausnitzii*, *Bacteroides thetaiotamicron, Holdemania filiformis*, *Dorea formicogenerans*	Beneficial	-
Kaderbhai et al. 47	PD-1	Antibiotic treatment	Nonbeneficial	-
Sivan et al. 48	PD-1	Bifidobacterium	Beneficial	Induce DC maturation and intratumor CD8+ T cell accumulation
Vetizou et al. 49	CTLA-4	*Bacteroides thetaiotaomicron*, *Bacteroides fragilis*	Beneficial	Elicit Th1 immune response and DC maturation
		*Burkholderia cepacia*	Beneficial	Synergize with TLR2/TLR4
Iida et al. 35	IL-10R plus CpG oligonucleotide	Alistipes, Ruminococcus	Beneficial	Activate tumor-infiltrating myeloid cells via TLR4 and increase TNF response
		*Lactobacillus fermentum*	Nonbeneficial	Decrease TNF response

The mechanisms refer to the underlined components when only a portion of the microbiota have been clarified. TLR, Toll-like receptor; CDD, cytidine deaminase; Tregs, regulatory T cells; Th, T helper; DC, dendritic cells; TNF, tumor necrosis factor.

## Abbreviations

5-FU: 5-Fluorouracil
*A. muciniphila*: *Akkermansia muciniphila*
*C. aerofaciens*: *Collinsella aerofaciens*
CDD: Cytidine deaminase
CRC: Colorectal cancer
CTX: Cyclophosphamide
DCs: Dendritic cells
dFdU: 2',2'-Difluorodeoxyuridine
*E. coli*: *Escherichia coli*
*E. faecium*: *Enterococcus faecium*
*E. hirae*: *Enterococcus hirae*
FDA: Food and Drug Administration
FMT: Fecal microbiota transplantation
FUDR: 5-Fluoro-2′-deoxyuridine
GEMM: Genetically engineered mouse model
ICIs: Immune checkpoint inhibitors
IL-12: Interleukin-12
IFN-γ: Interferon-gamma
*M. hyorhinis*: *Mycoplasma hyorhinis*
MDSCs: Myeloid-derived suppressor cells
PD-1: Programmed cell death protein 1
PDAC: Pancreatic ductal adenocarcinoma
rRNA: Ribosomal RNA
TAMs: Tumor-associated macrophages
Tc1: Cytotoxic T cell 1
Th1: T helper 1
TLRs: Toll-like receptors
TME: Tumor microenvironment
TNF: tumor necrosis factor
Treg: Regulatory T cell

## References

[1] Amrutkar M, Gladhaug IP (2017). Pancreatic cancer chemoresistance to gemcitabine. Cancers (Basel).

[2] de Sousa CL, Monteiro G (2014). Gemcitabine: metabolism and molecular mechanisms of action, sensitivity and chemoresistance in pancreatic cancer. Eur J Pharmacol.

[3] Banerjee K, Kumar S, Ross KA (2018). Emerging trends in the immunotherapy of pancreatic cancer. Cancer Lett.

[4] Bhatt AP, Redinbo MR, Bultman SJ (2017). The role of the microbiome in cancer development and therapy. CA Cancer J Clin.

[5] Zitvogel L, Daillere R, Roberti MP (2017). Anticancer effects of the microbiome and its products. Nat Rev Microbiol.

[6] Michaud DS, Joshipura K, Giovannucci E (2007). A prospective study of periodontal disease and pancreatic cancer in US male health professionals. J Natl Cancer Inst.

[7] Farrell JJ, Zhang L, Zhou H (2012). Variations of oral microbiota are associated with pancreatic diseases including pancreatic cancer. Gut.

[8] Chang JS, Tsai CR, Chen LT (2016). Investigating the association between periodontal disease and risk of pancreatic cancer. Pancreas.

[9] Heikkila P, But A, Sorsa T (2018). Periodontitis and cancer mortality: register-based cohort study of 68,273 adults in 10-year follow-up. Int J Cancer.

[10] Akshintala VS, Talukdar R, Singh VK (2019). The gut microbiome in pancreatic disease. Clin Gastroenterol Hepatol.

[11] Dauer P, Nomura A, Saluja A (2017). Microenvironment in determining chemo-resistance in pancreatic cancer: neighborhood matters. Pancreatology.

[12] Liu Q, Liao Q, Zhao Y (2017). Chemotherapy and tumor microenvironment of pancreatic cancer. Cancer Cell Int.

[13] Chand S, O'Hayer K, Blanco FF (2016). The landscape of pancreatic cancer therapeutic resistance mechanisms. Int J Biol Sci.

[14] Catenacci DV, Junttila MR, Karrison T (2015). Randomized phase Ib/II study of gemcitabine plus placebo or vismodegib, a *hedgehog* pathway inhibitor, in patients with metastatic pancreatic cancer. J Clin Oncol.

[15] Skelton RA, Javed A, Zheng L (2017). Overcoming the resistance of pancreatic cancer to immune checkpoint inhibitors. J Surg Oncol.

[16] Pu N, Lou W, Yu J (2019). PD-1 immunotherapy in pancreatic cancer: current status. J Pancreatol.

[17] Mei QX, Huang CL, Luo SZ (2018). Characterization of the duodenal bacterial microbiota in patients with pancreatic head cancer vs. healthy controls. Pancreatology.

[18] Maier L, Pruteanu M, Kuhn M (2018). Extensive impact of non-antibiotic drugs on human gut bacteria. Nature.

[19] Panebianco C, Adamberg K, Adamberg S (2017). Engineered resistant-starch (ERS) diet shapes colon microbiota profile in parallel with the retardation of tumor growth in *in vitro* and *in vivo* pancreatic cancer models. Nutrients.

[20] Alexander JL, Wilson ID, Teare J (2017). Gut microbiota modulation of chemotherapy efficacy and toxicity. Nat Rev Gastroenterol Hepatol.

[21] Yi M, Jiao D, Xu H (2018). Biomarkers for predicting efficacy of PD-1/PD-L1 inhibitors. Mol Cancer.

[22] Niess JH, Reinecker HC (2006). Dendritic cells in the recognition of intestinal microbiota. Cell Microbiol.

[23] Medzhitov R (2007). Recognition of microorganisms and activation of the immune response. Nature.

[24] Johansson ME, Phillipson M, Petersson J (2008). The inner of the two Muc2 mucin-dependent mucus layers in colon is devoid of bacteria. Proc Natl Acad Sci USA.

[25] Jakobsson HE, Rodriguez-Pineiro AM, Schutte A (2015). The composition of the gut microbiota shapes the colon mucus barrier. EMBO Rep.

[26] Zhang S, Yang Y, Weng W (2019). Fusobacterium nucleatum promotes chemoresistance to 5-fluorouracil by upregulation of BIRC3 expression in colorectal cancer. J Exp Clin Cancer Res.

[27] Yuan L, Zhang S, Li H (2018). The influence of gut microbiota dysbiosis to the efficacy of 5-fluorouracil treatment on colorectal cancer. Biomed Pharmacother.

[28] Deng X, Li Z, Li G (2018). Comparison of microbiota in patients treated by surgery or chemotherapy by 16S rRNA sequencing reveals potential biomarkers for colorectal cancer therapy. Front Microbiol.

[29] Geller LT, Barzily-Rokni M, Danino T (2017). Potential role of intratumor bacteria in mediating tumor resistance to the chemotherapeutic drug gemcitabine. Science.

[30] Yu T, Guo F, Yu Y (2017). Fusobacterium nucleatum promotes chemoresistance to colorectal cancer by modulating autophagy. Cell.

[31] Daillère R, Vétizou M, Waldschmitt N (2016). *Enterococcus hirae* and *Barnesiella intestinihominis* facilitate cyclophosphamide-induced therapeutic immunomodulatory effects. Immunity.

[32] An J, Ha EM (2016). Combination therapy of *Lactobacillus plantarum* supernatant and 5-fluouracil increases chemosensitivity in colorectal cancer cells. J Microbiol Biotechnol.

[33] Lehouritis P, Cummins J, Stanton M (2015). Local bacteria affect the efficacy of chemotherapeutic drugs. Sci Rep.

[34] Vande VJ, Sabuncuoglu S, Noppen S (2014). Nucleoside-catabolizing enzymes in mycoplasma-infected tumor cell cultures compromise the cytostatic activity of the anticancer drug gemcitabine. J Biol Chem.

[35] Iida N, Dzutsev A, Stewart CA (2013). Commensal bacteria control cancer response to therapy by modulating the tumor microenvironment. Science.

[36] Viaud S, Saccheri F, Mignot G (2013). The intestinal microbiota modulates the anticancer immune effects of cyclophosphamide. Science.

[37] Zheng Y, Wang T, Tu X (2019). Gut microbiome affects the response to anti-PD-1 immunotherapy in patients with hepatocellular carcinoma. J Immunother Cancer.

[38] Peters BA, Wilson M, Moran U (2019). Relating the gut metagenome and metatranscriptome to immunotherapy responses in melanoma patients. Genome Med.

[39] Zhao S, Gao G, Li W (2019). Antibiotics are associated with attenuated efficacy of anti-PD-1/PD-L1 therapies in Chinese patients with advanced non-small cell lung cancer. Lung Cancer.

[40] Matson V, Fessler J, Bao R (2018). The commensal microbiome is associated with anti-PD-1 efficacy in metastatic melanoma patients. Science.

[41] Gopalakrishnan V, Spencer CN, Nezi L (2018). Gut microbiome modulates response to anti-PD-1 immunotherapy in melanoma patients. Science.

[42] Pushalkar S, Hundeyin M, Daley D (2018). The pancreatic cancer microbiome promotes oncogenesis by induction of innate and adaptive immune suppression. Cancer Discov.

[43] Routy B, Le Chatelier E, Derosa L (2018). Gut microbiome influences efficacy of PD-1-based immunotherapy against epithelial tumors. Science.

[44] Derosa L, Hellmann MD, Spaziano M (2018). Negative association of antibiotics on clinical activity of immune checkpoint inhibitors in patients with advanced renal cell and non-small-cell lung cancer. Ann Oncol.

[45] Chaput N, Lepage P, Coutzac C (2017). Baseline gut microbiota predicts clinical response and colitis in metastatic melanoma patients treated with ipilimumab. Ann Oncol.

[46] Frankel AE, Coughlin LA, Kim J (2017). Metagenomic shotgun sequencing and unbiased metabolomic profiling identify specific human gut microbiota and metabolites associated with immune checkpoint therapy efficacy in melanoma patients. Neoplasia.

[47] Kaderbhai C, Richard C, Fumet JD (2017). Antibiotic use does not appear to influence response to nivolumab. Anticancer Res.

[48] Sivan A, Corrales L, Hubert N (2015). Commensal *Bifidobacterium* promotes antitumor immunity and facilitates anti-PD-L1 efficacy. Science.

[49] Vetizou M, Pitt JM, Daillere R (2015). Anticancer immunotherapy by CTLA-4 blockade relies on the gut microbiota. Science.

[50] Sousa T, Paterson R, Moore V (2008). The gastrointestinal microbiota as a site for the biotransformation of drugs. Int J Pharm.

[51] Hiroshima Y, Zhang Y, Murakami T (2014). Efficacy of tumor-targeting *Salmonella typhimurium* A1-R in combination with anti-angiogenesis therapy on a pancreatic cancer patient-derived orthotopic xenograft (PDOX) and cell line mouse models. Oncotarget.

[52] Paulos CM, Wrzesinski C, Kaiser A (2007). Microbial translocation augments the function of adoptively transferred self/tumor-specific CD8+ T cells via TLR4 signaling. J Clin Invest.

[53] Cogdill AP, Gaudreau PO, Arora R (2018). The impact of intratumoral and gastrointestinal microbiota on systemic cancer therapy. Trends Immunol.

[54] Ramos A, Hemann MT (2017). Drugs, bugs, and cancer: *Fusobacterium nucleatum* promotes chemoresistance in colorectal cancer. Cell.

[55] Garcia-Gonzalez AP, Ritter AD, Shrestha S (2017). Bacterial metabolism affects the *C. elegans* response to cancer chemotherapeutics. Cell.

[56] Montassier E, Gastinne T, Vangay P (2015). Chemotherapy-driven dysbiosis in the intestinal microbiome. Aliment Pharmacol Ther.

[57] Panebianco C, Adamberg K, Jaagura M (2018). Influence of gemcitabine chemotherapy on the microbiota of pancreatic cancer xenografted mice. Cancer Chemother Pharmacol.

[58] Conroy T, Hammel P, Hebbar M (2018). FOLFIRINOX or gemcitabine as adjuvant therapy for pancreatic cancer. N Engl J Med.

[59] Wallace BD, Wang H, Lane KT (2010). Alleviating cancer drug toxicity by inhibiting a bacterial enzyme. Science.

[60] Kurita A, Kado S, Matsumoto T (2011). Streptomycin alleviates irinotecan-induced delayed-onset diarrhea in rats by a mechanism other than inhibition of beta-glucuronidase activity in intestinal lumen. Cancer Chemother Pharmacol.

[61] Li R, Zhou R, Wang H (2019). Gut microbiota-stimulated cathepsin K secretion mediates TLR4-dependent M2 macrophage polarization and promotes tumor metastasis in colorectal cancer. Cell Death Differ.

[62] Chandra D, Jahangir A, Quispe-Tintaya W (2013). Myeloid-derived suppressor cells have a central role in attenuated *Listeria monocytogenes*-based immunotherapy against metastatic breast cancer in young and old mice. Br J Cancer.

[63] Lizotte PH, Baird JR, Stevens CA (2014). Attenuated *Listeria monocytogenes* reprograms M2-polarized tumor-associated macrophages in ovarian cancer leading to iNOS-mediated tumor cell lysis. Oncoimmunology.

[64] Round JL, Lee SM, Li J (2011). The toll-like receptor 2 pathway establishes colonization by a commensal of the human microbiota. Science.

[65] Sethi V, Kurtom S, Tarique M (2018). Gut microbiota promotes tumor growth in mice by modulating immune response. Gastroenterology.

[66] Tumeh PC, Harview CL, Yearley JH (2014). PD-1 blockade induces responses by inhibiting adaptive immune resistance. Nature.

[67] Spranger S, Luke JJ, Bao R (2016). Density of immunogenic antigens does not explain the presence or absence of the T-cell-inflamed tumor microenvironment in melanoma. Proc Natl Acad Sci USA.

[68] Trujillo JA, Sweis RF, Bao R (2018). T cell-inflamed versus non-T cell-inflamed tumors: a conceptual framework for cancer immunotherapy drug development and combination therapy selection. Cancer Immunol Res.

[69] Yi M, Yu S, Qin S (2018). Gut microbiome modulates efficacy of immune checkpoint inhibitors. J Hematol Oncol.

[70] Komai-Koma M, Wang E, Kurowska-Stolarska M (2016). Interleukin-33 promoting Th1 lymphocyte differentiation dependents on IL-12. Immunobiology.

[71] Thomas H (2017). Pancreatic cancer: intra-tumour bacteria promote gemcitabine resistance in pancreatic adenocarcinoma. Nat Rev Gastroenterol Hepatol.

[72] del Castillo E, Meier R, Chung M (2019). The microbiomes of pancreatic and duodenum tissue overlap and are highly subject specific but differ between pancreatic cancer and noncancer subjects. Cancer Epidemiol Biomarkers Prev.

[73] Louis P, Hold GL, Flint HJ (2014). The gut microbiota, bacterial metabolites and colorectal cancer. Nat Rev Microbiol.

[74] Amieva M, Peek RM (2016). Pathobiology of *Helicobacter pylori*-induced gastric cancer. Gastroenterology.

[75] Koshiol J, Wozniak A, Cook P (2016). *Salmonella enterica* serovar typhi and gallbladder cancer: a case-control study and meta-analysis. Cancer Med.

[76] Aziz RK, Hegazy SM, Yasser R (2018). Drug pharmacomicrobiomics and toxicomicrobiomics: from scattered reports to systematic studies of drug-microbiome interactions. Expert Opin Drug Metab Toxicol.

[77] Avan A, Caretti V, Funel N (2013). Crizotinib inhibits metabolic inactivation of gemcitabine in c-Met-driven pancreatic carcinoma. Cancer Res.

[78] Mitsuhashi K, Nosho K, Sukawa Y (2015). Association of *Fusobacterium* species in pancreatic cancer tissues with molecular features and prognosis. Oncotarget.

[79] Kelly MG, Alvero AB, Chen R (2006). TLR-4 signaling promotes tumor growth and paclitaxel chemoresistance in ovarian cancer. Cancer Res.

[80] Delitto D, Delitto AE, Di Vita BB (2017). Human pancreatic cancer cells induce a MyD88-dependent stromal response to promote a tumor-tolerant immune microenvironment. Cancer Res.

[81] Liu Q, Li Y, Niu Z (2016). Atorvastatin (Lipitor) attenuates the effects of aspirin on pancreatic cancerogenesis and the chemotherapeutic efficacy of gemcitabine on pancreatic cancer by promoting M2 polarized tumor associated macrophages. J Exp Clin Cancer Res.

[82] Riquelme E, Maitra A, McAllister F (2018). Immunotherapy for pancreatic cancer: more than just a gut feeling. Cancer Discov.

[83] Riquelme E, Zhang Y, Zhang L (2019). Tumor microbiome diversity and composition influence pancreatic cancer outcomes. Cell.

[84] Zhang F, Cui B, He X (2018). Microbiota transplantation: concept, methodology and strategy for its modernization. Protein Cell.

[85] Vrieze A, Van Nood E, Holleman F (2012). Transfer of intestinal microbiota from lean donors increases insulin sensitivity in individuals with metabolic syndrome. Gastroenterology.

[86] Kao D, Roach B, Park H (2016). Fecal microbiota transplantation in the management of hepatic encephalopathy. Hepatology.

[87] He Z, Cui B-T, Zhang T (2017). Fecal microbiota transplantation cured epilepsy in a case with Crohn's disease: the first report. World J Gastroenterol.

[88] Le Bastard Q, Ward T, Sidiropoulos D (2018). Fecal microbiota transplantation reverses antibiotic and chemotherapy-induced gut dysbiosis in mice. Sci Rep.

[89] Wang Y, Wiesnoski DH, Helmink BA (2018). Fecal microbiota transplantation for refractory immune checkpoint inhibitor-associated colitis. Nat Med.

[90] Tanoue T, Morita S, Plichta DR (2019). A defined commensal consortium elicits CD8 T cells and anti-cancer immunity. Nature.

[91] Nelson MH, Diven MA, Huff LW (2015). Harnessing the microbiome to enhance cancer immunotherapy. J Immunol Res.

[92] Pitt JM, Vétizou M, Boneca IG (2016). Enhancing the clinical coverage and anticancer efficacy of immune checkpoint blockade through manipulation of the gut microbiota. Oncoimmunology.

[93] Touchefeu Y, Montassier E, Nieman K (2014). Systematic review: the role of the gut microbiota in chemotherapy- or radiation-induced gastrointestinal mucositis - current evidence and potential clinical applications. Aliment Pharmacol Ther.

[94] van Vliet MJ, Harmsen HJ, de Bont ES (2010). The role of intestinal microbiota in the development and severity of chemotherapy-induced mucositis. PLoS Pathog.

[95] Adachi K, Tamada K (2018). Microbial biomarkers for immune checkpoint blockade therapy against cancer. J Gastroenterol.

[96] Zimmermann M, Zimmermann-Kogadeeva M, Wegmann R (2019). Separating host and microbiome contributions to drug pharmacokinetics and toxicity. Science.

[97] Clayton TA, Baker D, Lindon JC (2009). Pharmacometabonomic identification of a significant host-microbiome metabolic interaction affecting human drug metabolism. Proc Natl Acad Sci USA.

[98] Panebianco C, Andriulli A, Pazienza V (2018). Pharmacomicrobiomics: exploiting the drug-microbiota interactions in anticancer therapies. Microbiome.

[99] Forbes NS, Coffin RS, Deng L (2018). White paper on microbial anti-cancer therapy and prevention. J Immunother Cancer.

[100] Klement RJ, Pazienza V (2019). Impact of different types of diet on gut microbiota profiles and cancer prevention and treatment. Medicina (Kaunas).

[101] Panebianco C, Potenza A, Andriulli A (2018). Exploring the microbiota to better understand gastrointestinal cancers physiology. Clin Chem Lab Med.

